# Chemotherapy enriches for an invasive triple-negative breast tumor cell subpopulation expressing a precursor form of N-cadherin on the cell surface

**DOI:** 10.18632/oncotarget.12767

**Published:** 2016-10-28

**Authors:** Erik R. Nelson, Shenduo Li, Margaret Kennedy, Sturgis Payne, Kelly Kilibarda, Jeffrey Groth, Michelle Bowie, Edgardo Parilla-Castellar, Gustaaf de Ridder, Paul Kelly Marcom, Matthew Lyes, Bercedis L. Peterson, Michael Cook, Salvatore V. Pizzo, Donald P. McDonnell, Robin E. Bachelder

**Affiliations:** ^1^ Department of Molecular and Integrative Physiology, University of Illinois at Urbana Champaign, Urbana and University of Illinois Cancer Center, University of Illinois at Chicago, Chicago, IL, USA; ^2^ Department of Biostatistics and Bioinformatics, Duke University School of Medicine, Durham, NC, USA; ^3^ Department of Immunology, Duke University School of Medicine, Durham, NC, USA; ^4^ Department of Pathology, Duke University Medical Center, Durham, NC, USA; ^5^ Department of Pharmacology and Cancer Biology, Duke University Medical Center, Durham, NC, USA

**Keywords:** Precursor-N-cadherin, triple-negative breast cancer, invasion, metastasis, chemotherapy resistance

## Abstract

**Background:**

Although most triple-negative breast cancer (TNBC) patients initially respond to chemotherapy, residual tumor cells frequently persist and drive recurrent tumor growth. Previous studies from our laboratory and others' indicate that TNBC is heterogeneous, being composed of chemo-sensitive and chemo-resistant tumor cell subpopulations. In the current work, we studied the invasive behaviors of chemo-resistant TNBC, and sought to identify markers of invasion in chemo-residual TNBC.

**Methods:**

The invasive behavior of TNBC tumor cells surviving short-term chemotherapy treatment *in vitro* was studied using transwell invasion assays and an experimental metastasis model. mRNA expression levels of neural cadherin (N-cadherin), an adhesion molecule that promotes invasion, was assessed by PCR. Expression of N-cadherin and its precursor form (pro-N-cadherin) was assessed by immunoblotting and flow cytometry. Pro-N-cadherin immunohistochemistry was performed on tumors obtained from patients pre- and post- neoadjuvant chemotherapy treatment.

**Results:**

TNBC cells surviving short-term chemotherapy treatment exhibited increased invasive behavior and capacity to colonize metastatic sites compared to untreated tumor cells. The invasive behavior of chemo-resistant cells was associated with their increased cell surface expression of precursor N-cadherin (pro-N-cadherin). An antibody specific for the precursor domain of N-cadherin inhibited invasion of chemo-resistant TNBC cells. To begin to validate our findings in humans, we showed that the percent cell surface pro-N-cadherin (+) tumor cells increased in patients post- chemotherapy treatment.

**Conclusions:**

TNBC cells surviving short-term chemotherapy treatment are more invasive than bulk tumor cells. Cell surface pro-N-cadherin expression is associated with the invasive and chemo-resistant behaviors of this tumor cell subset. Our findings indicate the importance of future studies determining the value of cell surface pro-N-cadherin as: 1) a biomarker for TNBC recurrence and 2) a therapeutic target for eliminating chemo-residual disease.

## INTRODUCTION

Most triple-negative breast cancers respond initially to chemotherapy. However, residual tumor cells frequently persist. These residual tumor cells are thought to be responsible for recurrent tumor growth (local and distant), which frequently occurs within 3 years of treatment [1], accounting for the high mortality rate of this breast cancer subtype. The clinically unmet need for better therapeutic approaches to treat this disease underscores the importance of characterizing the signaling pathways in residual tumor cells that drive tumor recurrence post-therapy.

It is now well-appreciated that tumors are heterogeneous, being composed of chemotherapy-sensitive and chemotherapy-resistant tumor cell subpopulations [2, 3]. Because the resistant subpopulations are frequently under-represented in the tumor bulk, the identification of markers and/or behaviors of chemo-resistant subpopulations has proven elusive. Several studies indicate that chemo-resistance is associated with cancer stem-like cell behaviors [4-8]. However, the relevance of cancer stem cell-like populations to TN breast cancer recurrence remains controversial.

Previously we described a method for studying TN breast cancer cell subpopulations enriched by short-term chemotherapy treatment [9]. In this model, short-term chemotherapy treatment of TN breast tumor cells enriches for chemo-resistant, growth-arrested tumor cells. These chemo-residual tumor cells resume growth after removing the chemotherapeutic agent, and subsequently establish drug resistant colonies [9]. This model resembles the clinical setting of a chemotherapeutic “rest period” or “drug holiday”, which occurs between chemotherapy cycles [10]. Colonies emanating from chemo-residual tumor cells after chemotherapy removal resemble recurrent tumors in that they exhibit multidrug resistance [9]. In the current work, we show that chemo-resistant TN breast tumor cells emanating from this short-term chemotherapy treatment model exhibit increased invasive/metastatic behavior. Our findings suggest that chemotherapy drives the evolution of more aggressive TN breast cancers by enriching for a highly invasive tumor cell sub-population. Moreover, , we show that these chemotherapy-enriched, aggressive tumor cell subpopulations do not exhibit classic properties of cancer stem-like cells. Finally, we identify a novel adhesion marker expressed on the surface of chemo-resistant TN tumor cells that drives their invasive phenotype, and demonstrate that this marker is increased in primary TN breast cancers post neoadjvuant chemotherapy treatment.

## RESULTS

**Figure 1:**
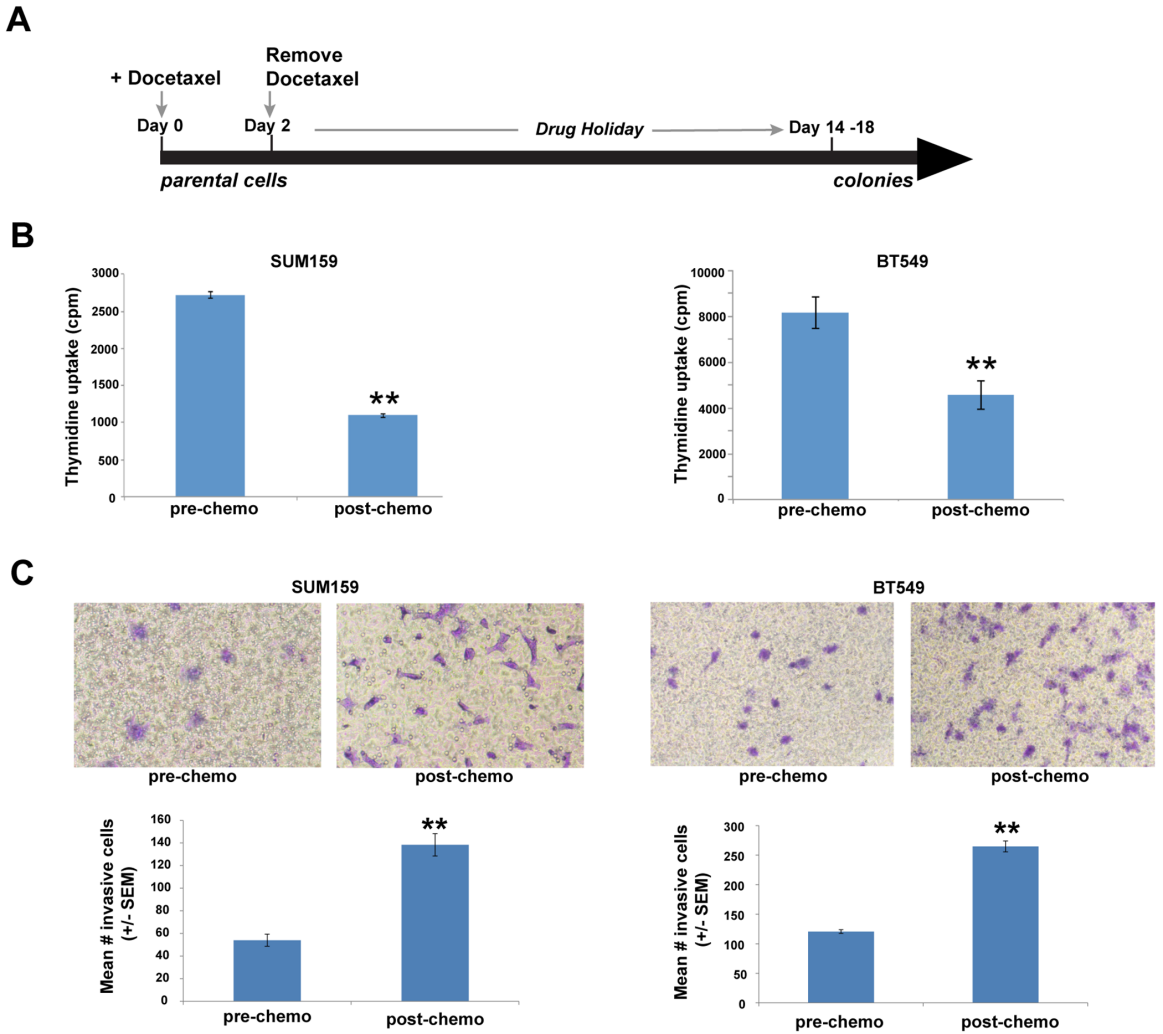
Chemo-residual triple-negative (TN) breast tumor cells emanating from short-term chemotherapy treatment model exhibit increased invasive phenotype **A.** SUM159 and BT549 tumor cells were exposed to docetaxel (100 nM) for 2 d, after which drug was removed. On d8, only a sub-population of chemo-residual cells remained. Approximately two weeks after chemotherapy withdrawal, these cells resumed growth, establishing colonies. **B.** Relative proliferative potential of parental and chemo-residual tumor cells (harvested on d18) was measured in a thymidine uptake assay. Results are reported as mean thymidine uptake from six wells (+/- SEM) for each cell population. Note that chemo-residual tumor cells exhibited reduced proliferation compared to parental tumor cells. **, SUM159, *p* = 5.5 x 10^-11^; **, BT549, *p* = 0.0001. **C.** Invasive potential of parental and chemo-residual SUM159 (left panel)****and BT549 (right panel) tumor cells was measured in a Matrigel transwell assay. Top panel shows a representative field of crystal-violet stained invasive cells (100X magnification). Bottom panel shows quantitation of invasion, determined by counting the mean # invasive cells from triplicate wells [+/- standard error of the mean (SEM)] for each of the cell populations. Similar results were obtained in at least 3 independent trials for **A.**-**C.** **, SUM159; *p* = 0.01; **, BT549- *p* = 0.005, *t*-test.

We have developed a short-term chemotherapy treatment model that enriches for a chemo-resistant subset of TN breast tumor cells [9] (Figure 1A). In this model, triple-negative breast cancer cells (SUM159, BT549) were exposed to a clinically-relevant chemotherapy regimen (docetaxel) for two days, after which drug was removed from the medium. By day 7, we observed a subpopulation of growth-arrested tumor cells surviving chemotherapy. Approximately 2 weeks after chemotherapy removal, this chemo-residual tumor cell subpopulation resumed growth, establishing colonies (Figure 1A). In our previous work, we showed that colonies emanating from this short-term chemotherapy-treatment model exhibit multi-drug resistance (9). In the current work, we investigated the invasive potential of chemo-resistant TN breast tumor colonies established after chemotherapy removal. As shown in Figure 1B, colonies evolving from this short-term docetaxel treatment model exhibited reduced proliferation compared to untreated parental cells. Notably, chemo-resistant TN tumor cells arising from this model also exhibited significantly increased invasive potential, as measured in a matrigel transwell assay (Figure 1C).

**Figure 2:**
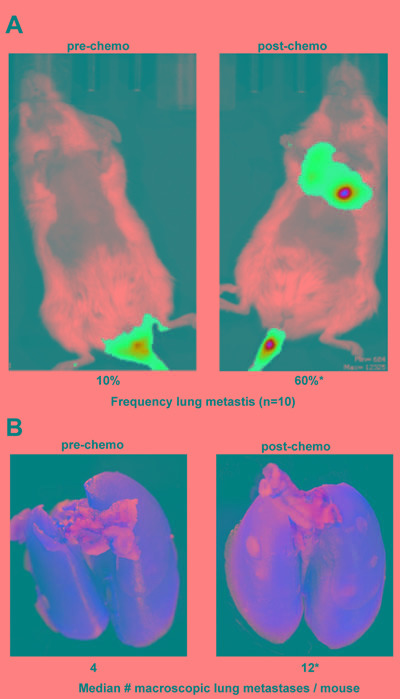
Chemo-residual TN breast tumor cells exhibit increased lung colonization Luciferase-expressing parental and chemo-residual SUM159 cells (harvested on d18 as in Figure 1) were injected into the tail vein of NSG mice (ten mice per group). **A.** On d33, luciferase-expressing lung colonies were visualized by luminescence (left panel). Frequency of lung colonization for each group (*n* = 10), was assessed by luciferase signal, is indicated. *, *p* = 0.03. **B.** After 34 d, animals were sacrificed, and lungs were removed and photographed (left panel). Macro-metastases were counted, and are reported as median number macroscopic metastases/mouse.

Based on their increased invasive phenotype, we next sought to determine if these chemo-resistant tumor cells, when injected into the tail vein of immunocompromised mice, exhibited increased ability to colonize the lung compared to untreated tumor cells. First, luciferase-expressing SUM159 TN tumor cells were subjected to short-term docetaxel treatment as in Figure 1A, after which chemotherapy was removed. Colonies emanating from this model on day 18 were harvested, and their lung colonizing potential was measured in a tail vein injection model using NOD scid gamma (NSG) mice. Specifically, NSG mice were divided into two groups (10 mice/group). The first group was injected with luciferase-expressing parental SUM159 TN tumor cells (Pre-chemo). The second group was injected with luciferase-expressing chemo-residual SUM159 cells (Post-chemo). On day 33, the luciferase signal in the lung was determined by bioluminescence. Strikingly, TN tumor cells obtained post-chemotherapy treatment colonized the lung in six of ten mice (60%) whereas parental TN tumor cells colonized the lung in only one of ten mice (10%) (Figure 2A). The increased frequency of lung colonization by chemo-resistant TN tumor cells was observed despite the fact that the chemo-resistant tumor cells exhibited a lower luciferase signal/cell than parental tumor cells (Supplementary Figure 1). On day 34, animals were sacrificed, lungs were harvested, and the number of macroscopic lung metastases/mouse was determined. Lungs from mice grafted with chemo-resistant SUM159 cells emanating from our short-term chemotherapy treatment model exhibited an increased number of macroscopic lung metastases/mouse compared to those from mice grafted with parental SUM159 cells (Figure 2B).

Previous studies indicate that long-term chemotherapy selection models drive the growth of cancer stem-like cells [4-8]. We therefore sought to determine if chemo-resistant TN tumor cells emanating from our short-term chemotherapy treatment model exhibit cancer stem-like properties. As shown in Supplementary Figure 2, chemo-resistant tumor cells from our model did not exhibit an increased ability to grow as non-adherent spheres, a defining property of cancer stem-like cells. In fact, they had decreased ability compared to their non-treated parental counterparts. To measure their self-renewing activity, primary spheres were dissociated into single cells, and the efficiency of secondary sphere formation was determined. As shown in Suppl. Figure 2B, chemo-resistant tumor cells from our model did not exhibit increased self-renewing activity compared to parental tumor cells. Because cancer stem-like cells exhibit increased tumor-initiating activity, we next assessed the relative tumor-initiating ability of chemo-resistant and parental triple-negative tumor cells in an orthotopic mouse model. SUM159 cells obtained pre- and post-chemotherapy were injected in a limiting dilution study into the mammary fat pad of NSG mice (10 mice/group). Tumor volumes were assessed using calipers on a weekly basis until tumors reached a size of 100 mm^3^, at which point they were measured every 2-3 days until volumes reached 2000 mm^3^. As shown in Suppl. Figure 2C, tumor cells obtained post-chemotherapy treatment did not exhibit increased tumor-initiating activity compared to untreated TN tumor cells at any injection number. Furthermore, there were no differences in tumor growth rate between chemo-residual and parental grafts (Supplementary Figure 3).

**Figure 3:**
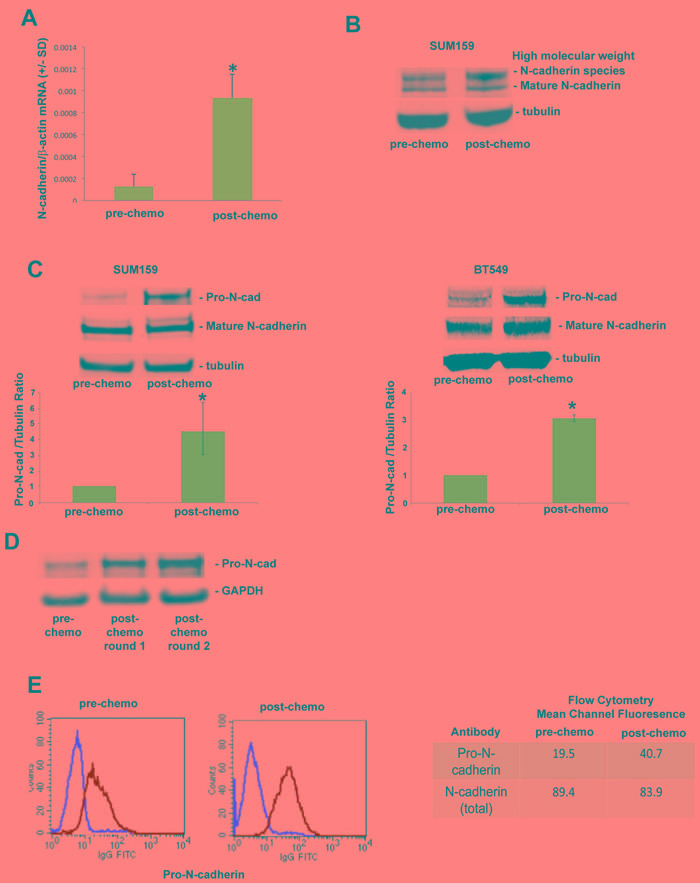
Precursor (pro) form of N-cadherin is upregulated on the cell surface of chemo-residual TN tumor cells **A.** mRNA was isolated from parental and chemo-residual SUM159 tumor cells (harvested on d18, as in Figure 1A). N-cadherin and beta actin levels were determined by quantitative real time PCR. Data are reported as the ratio of N-cadherin/beta actin (+/- SD from three trials). *, *p* = 0.05, *t*-test. **B.** Total cell extracts were obtained from EDTA-detached parental and chemo-residual SUM159 tumor cells. Equivalent amounts were immunoblotted with an N-cadherin antibody, followed by the appropriate IRdye-labelled secondary antibody. Protein bands were detected by Odyssey infrared imaging. Similar results were observed in 4 independent trials. Note the presence of increased levels of a high molecular weight N-cadherin species in chemo-residual cells compared to parental cells. **C.** Total cell extracts were obtained from SUM159 and BT549 parental and chemo-residual tumor cells described in Figure 1. In the top panels, equivalent amounts of protein were immunoblotted with pro-N-cadherin, N-cadherin, or Tubulin antibody, followed by IRDye conjugated secondary antibody. Similar results were obtained in three independent experiments. Bottom panels show the ratio of Pro-N-cadherin to Tubulin from three independent trials. SUM159 *, *p* = 0.05, *t*-test. BT549 *, *p* = 0.03. **D.** Chemo-residual SUM159 tumor cells emanating from our model were subjected to a second round of short-term docetaxel (100 nM) treatment using the same methods as described in Figure 1. Pro-N-cadherin expression levels in chemo-resistant tumor cells generated after one or two rounds of docetaxel treatment were assessed as described in **C.** **E.** Parental and chemo-residual SUM159 tumor cells (generated in Figure 1A) were harvested with EDTA (+/- SD), stained with a pro-N-cadherin or N-cadherin antibody, followed by FITC-conjugated secondary antibody, and analyzed by flow cytometry. Histograms are shown in the left panel. Intensity of staining is indicated as mean channel fluorescence in the right panel. Similar results were obtained in three independent trials.

Long-term chemotherapy selection models drive an epithelial-mesenchymal transition in estrogen receptor-positive breast tumors, characterized by reduced epithelial adhesion marker (E-cadherin) and acquired mesenchymal adhesion marker (N-cadherin) expression. By contrast, triple-negative breast cancers are typically mesenchymal in nature, expressing significant N-cadherin prior to chemotherapy treatment. We performed real-time PCR to determine relative levels of N-cadherin in parental (untreated) and chemo-resistant SUM159 cells from our short term chemotherapy treatment model. As shown in Figure 3A, SUM159 cells obtained post-chemotherapy treatment exhibited a seven-fold increase in N-cadherin mRNA levels compared to that observed in untreated SUM159 cells. Surprisingly, levels of N-cadherin protein (120 kDa) were equal in in SUM159 cells obtained pre- and post-chemotherapy treatment (Figure 3B). We did however observe that the N-cadherin antibody reacted with a higher molecular weight species, the expression of which was significantly increased in SUM159 tumor cells obtained post-chemotherapy treatment compared to parental SUM159 tumor cells (Figure 3B). Based on the knowledge that N-cadherin is synthesized as a precursor protein (pro-N-cadherin) that is cleaved by proteases to generate the mature form [11], we next investigated levels of pro-N-cadherin in TN tumor cells obtained pre- and post-chemotherapy treatment. Chemo-resistant SUM159 and BT549 cells generated in our short term chemotherapy treatment model expressed significantly increased levels of Pro-N-cadherin compared to untreated cells, as detected using an antibody specific for this precursor N-cadherin protein (Figure 3C). Notably, pro-N-cadherin protein levels were equal in chemo-resistant SUM159 cells exposed to either one or two rounds of short-term docetaxel treatment (Figure 3D), indicating that pro-N-cadherin expression was maintained over time in chemo-resistant cells.

In untransformed cells, only the mature form of N-cadherin, and not the precursor protein, is transported to the cell surface [11]. By contrast, recent studies indicate that pro-N-cadherin itself can be transported to the surface of tumor cells, driving an invasive phenotype [12]. Accordingly, we next investigated cell surface expression of Pro-N-cadherin in chemo-resistant and parental TN tumor cells by flow cytometry. Tumor cells obtained post-chemotherapy treatment expressed significantly increased levels of cell surface pro-N-cadherin compared to untreated tumor cells, as reflected by a 2-fold increase in the mean channel fluorescence (Figure 3E).

**Figure 4:**
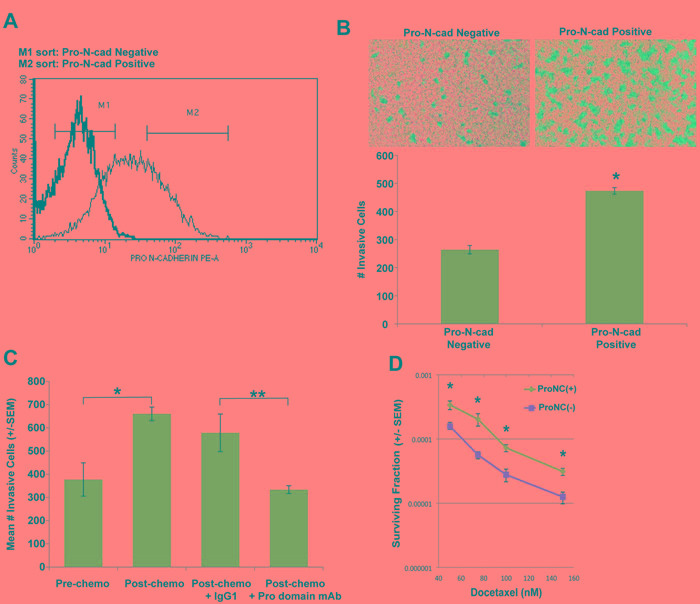
A sub-population of TN tumor cells expressing cell surface pro-N-cadherin exhibits increased invasive behavior **A.** SUM159 cells were stained with a Pro-N-cadherin antibody (faint line) or an isotype control antibody (bold line). Pro-N-cadherin-positive (M2) and pro-N-cadherin-negative (M1) SUM159 tumor cells were isolated by cell sorting. **B.** Invasive potential of pro-N-cadherin- sorted TN tumor cell subpopulations was determined in matrigel-coated transwells as in Figure 1C. Top panel shows a representative field of crystal violet stained invasive cells (100 x magnification). Bottom panel shows quantitation of invasion, determined by counting the mean # invasive cells from triplicate wells (+/- SEM). Similar results were obtained in two independent trials. *, *p* = 0.01, *t*-test. **C.** Parental and chemo-residual SUM159 cells were placed in matrigel-coated Transwell chambers for 4 h +/- monoclonal antibody specific for the N-cadherin precursor domain (Pro domain mAb; 10A10) [30] or isotype control antibody (IgG1) at a concentration of 5 μg/mL. Mean # invasive cells from triplicate wells (+/- SEM) was determined. Similar results were obtained in three independent trials. *, *p* = 0.03, ** *p* = 0.007. **D.** Pro-N-cadherin-positive and pro-N-cadherin-negative SUM159 sorted cells were exposed to the indicated docetaxel concentrations and surviving fraction was determined in a clonogenic assay. Mean # colonies from three wells (+/- SEM) was determined for each cell population. The *t*-test was implemented to determine statistically significantly differences in surviving fraction for the two sorted populations at each docetaxel concentration. * 50 nM, *p* = 0.04; * 75 nM, *p* = 0.02; * 100 nM, *p* = 0.01; * 150 nM, *p* = 0.01.

Pro-N-cadherin is expressed on the surface of melanoma and glioma cells, and contributes to their invasive behavior [12]. We hypothesized that the short-term chemotherapy treatment enriches for a resistant TN breast tumor cell subpopulation expressing cell surface pro-N-cadherin. To test this hypothesis, we performed cell sorting on untreated SUM159 TN tumor cells to separate cell-surface pro-N-cadherin-positive from cell-surface pro-N-cadherin-negative tumor cells (Figure 4A), and investigated the relative invasive potential of these sorted populations. As shown in Figure 4B, cell surface Pro-N-cadherin-positive SUM159 cells exhibited an approximately two-fold increase in transwell invasion compared to cell surface-Pro-N-cadherin-negative SUM159 cells. These data demonstrate that a subpopulation of SUM159 tumor cells expressing cell surface pro-N-cadherin exhibits increased invasive behavior relative to the subpopulation lacking this protein. To directly link cell surface pro-N-cadherin to the invasive behavior of chemo-resistant TN breast tumor cells, we showed that incubation of SUM159 tumor cells obtained post-chemotherapy treatment with an antibody specific for the precursor (pro) domain of N-cadherin significantly reduced their transwell invasion (Figure 4C).

Based on the knowledge that the cell surface pro-N-cadherin- positive population was enriched by chemotherapy treatment of SUM159 cells (Figure 4), we next investigated the relative chemo-resistance of pro-N-cadherin-positive *vs* pro-N-cadherin-negative sorted populations from untreated SUM159 tumor cells. As shown in Figure 4D, the pro-N-cadherin-positive population was significantly more resistant to docetaxel than the pro-N-cadherin-negative sorted population. Collectively, these data indicate that: 1) a highly invasive and chemo-resistant subpopulation of TN tumor cells expresses high levels of cell surface pro-N-cadherin, and 2) this subpopulation is enriched by short-term chemotherapy treatment.

We next sought to validate our findings in chemo-residual tumor cells from TN breast cancer patients. Matched tumor biopsies were obtained from TN breast cancer patients (*n* = 6 cases) pre- and post- neoadjuvant chemotherapy treatment. Pro-N-cadherin expression levels were assessed in these tumor tissues by immunohistochemistry. Notably, we observed nuclear/peri-nuclear pro-N-cadherin in both pre- and post-chemotherapy cases (Supplementary Figure 4; white arrows). In contrast, in the majority of post-chemotherapy cases, we detected cell membrane pro-N-cadherin staining (Supplementary Figure 4; black arrows). Specimens were scored in a blinded fashion by two pathologists for % tumor cells expressing cell membrane (surface) Pro-N-cadherin, as well as intensity of staining (Table 1). For most of the cases, cell surface Pro-N-cadherin was detected in less than 5% of the tumor cells of tissues obtained pre-chemotherapy. In five of the six cases, the percentage of tumor cells expressing cell surface pro-N-cadherin increased appreciably post-treatment (Table 1). These data: 1) support our *in vitro* findings, which indicate that a population of cell surface pro-N-cadherin-expressing cells is enriched by chemotherapy treatment, and 2) underscore the clinical significance of our results.

**Table 1:** Cell surface Pro-N-cadherin expression in triple-negative breast tumors pre- and post- neoadjuvant chemotherapy treatment

TNBC Case	Chemotherapy	% cell surface pro-N-cad(+) tumor cells pre-chemo	% cell surface Pro-N-cad(+) tumor cells post-chemo	Trend (Post vs Pre)
1	TCx4 + Ax4	0	50	↑
2	ACx4 + Pacx1	20	50	↑
3	TACx6	1	60	↑
4	ACx4 + Pacx4	3	40	↑
5	ACx4 + Pacx4	0	5	↑
6	ACx4 + Pacx1	5	0	↓

Six triple-negative breast cancer cases exhibiting an incomplete pathologic response to neoadjuvant chemotherapy were identified from medical records under Duke Institutional Review Board approval (Protocol 47289). Pro-N-cadherin expression in formalin-fixed, paraffin embedded tissues was assessed by immunohistochemistry using Pro-N-cadherin antibody (R&D Systems). Cell surface Pro-N-cadherin scoring was performed in a blinded fashion by two pathologists. Consensus scores for % cell surface Pro-N-cadherin(+) tumor cells are shown. Five of six cases showed increased % cell surface Pro-N-cadherin(+) tumor cells post-chemotherapy (↑). One of six cases showed reduced % cell surface Pro-N-cad(+) tumor cells post-chemotherapy (↑). Intensity of staining for all positive cases was +1.

## DISCUSSION

Hormone and receptor-targeted therapies are not available for TN breast cancers, which lack ER, PR and HER2 expression. Accordingly, chemotherapy is the only available treatment for women diagnosed with these aggressive tumors. Although TN tumors initially respond to chemotherapy, the response is incomplete in the majority of cases. Half of the women with an incomplete response will experience tumor recurrence within three years. Therefore, in order to develop effective therapies that reduce patient mortality, it is of the utmost importance to identify molecular determinants of TN chemo-residual disease that contribute to tumor recurrence.

 Continuous, long-term chemotherapy selection models promote the growth of cancer stem-like cells [8, 13]. Likewise, chemo-residual TN tumor cells surviving 4 days paclitaxel treatment exhibit cancer stem-like cell behaviors [4, 6]. Based on these results, we were surprised to find that TN tumor cells from our short-term chemotherapy treatment model did not exhibit cancer stem-like cell behaviors (Suppl. Figure 3). We propose two possible explanations for these discrepant results. First, the chemotherapy concentrations utilized in our studies (100-fold IC_50_) have been achieved in patients [14], but are significantly higher than the chemotherapy concentrations previously shown to promote growth of cancer stem-like cells (50% IC_50_) [4, 6]. Accordingly, we acknowledge the possibility that high but not low chemotherapy concentrations may eliminate cancer stem cells. Alternatively, cancer stem-like cells exhibit plasticity, with micro-environmental influences such as hypoxia being important for cancer stem cell maintenance [15, 16]. Thus, it is possible that maintenance of stem-like tumor cells is dependent on continuous chemotherapy exposure, contrasting with the conditions in our short-term chemotherapy treatment model.

Our studies are the first to show that chemotherapy enriches for a highly invasive tumor cell subpopulation, the maintenance of which is not dependent on continuous drug treatment. Of note, the evolution of these therapy-resistant breast tumor cells with increased invasive behavior was dependent on tumor cell exposure to high chemotherapy concentrations (100-fold IC_50_), which eliminated 50% of the tumor cells within 2 days (data not shown). Chemotherapy removal in our model mimics the “drug holiday” that cancer patients experience after initial treatment. Our results suggest that this drug holiday may allow for the expansion of a TN tumor cell subset that is both drug resistant and highly invasive.

Our demonstration that chemotherapy selects for an aggressive tumor cell population illustrates the previously described model of oncogenic resistance [17, 18]. According to this model, drugs select for tumor cells expressing oncoproteins that drive both resistance and aggressive behaviors contributing to tumor progression. Our studies describe a novel protein in therapy-resistant triple-negative breast tumor cells (pro-N-cadherin) that is associated with these behaviors.

Results from our study expand upon previous work, which demonstrated that cancer therapeutics can promote tumor progression by influencing the microenvironment [19, 20]. In these mouse models, angiogenesis inhibitors (*e.g.,* Sunitinib, anti-VEGFR2) were shown to establish a metastatic niche that promotes tumor metastasis [19, 20]. Notably, in these studies, the invasive tumors elicited by anti-angiogenic therapy remained invasive, even after withdrawing therapy. In another study, chemotherapy treatment of non-tumor bearing mice established a metastatic niche that enabled injected tumors to metastasize more efficiently than that which occurs in untreated mice [21]. Our studies add to these findings by showing that, in the absence of micro-environmental influences, chemotherapy drives triple-negative tumor cell invasive behavior by selecting for highly invasive tumor cell subpopulations represented infrequently in the heterogeneous tumor and characterized by the expression of cell surface pro-N-cadherin. Similar to the pro-metastatic activities of angiogenesis inhibitors reported previously, chemotherapy-driven tumor cell invasive behavior was not dependent on continuous chemotherapy treatment. Future studies are needed to determine if chemotherapy effects on tumor cells and chemotherapy effects on the tumor microenvironment are additive in driving tumor invasive/metastatic behavior.

Recent molecular profiling analyses identified novel markers of triple-negative breast cancer chemo-resistance by studying genes differentially expressed in patient tumors obtained pre- and post- neoadjuvant chemotherapy treatment [22, 23]. One drawback of molecular profiling analyses is that they fail to identify determinants of resistance that are regulated at post-transcriptional or post-translational levels. In the current work, we identify a precursor form of an adhesion molecule, the expression of which is increased in chemo-residual TN tumor cells compared to untreated tumors. Cell sorting studies indicate that cells expressing high levels of this precursor protein represent a pre-existing subpopulation in the original tumor cell line that is chemo-resistant (Figure 5C) and exhibits highly invasive behavior (Figure 5B). Because the expression level of this marker is determined by post-translational processing, DNA/RNA profiling methods would not identify this differentially expressed protein in chemo-residual tumor cells.

During the epithelial-mesenchymal transition, epithelial tumor cells undergo a cadherin-switch, losing expression of the epithelial adhesion marker E-cadherin and gaining expression of neural cadherin (N-cadherin). Acquired N-cadherin expression in these cells drives invasive and metastatic tumor cell behaviors [24-26]. N-cadherin is expressed as a precursor form (pro-N-cadherin) lacking adhesive function. A specific pro-protein convertase cleaves pro-N-cadherin in the Golgi apparatus, allowing for the adhesion molecule to be transported to the cell surface [11]. A recent study indicates that tumor cells (*e.g.*, melanoma, brain) exhibit a unique ability to transport pro-N-cadherin, the immature form of N-cadherin, to the cell surface [12]. Notably, cell surface-expressed pro-N-cadherin drives tumor cell invasion, and pro-N-cadherin expression is directly associated with breast cancer grade [12]. Our study adds to these findings by showing that cell surface pro-N-cadherin expression is detected in triple-negative breast cancers. Furthermore, we demonstrate that triple-negative breast tumors are heterogeneous, being composed of both cell surface pro-N-cadherin-positive and -negative tumor cell subpopulations. Finally, by cell sorting, we show that TN tumor cell subsets expressing cell surface pro-N-cadherin exhibit increased invasive behavior compared to TN tumor cells lacking this precursor protein on the cell surface.

Our studies of TN breast cancer cases (*n* = 6) obtained pre- and post- neoadjuvant chemotherapy treatment demonstrate that most patients exhibit an increased percentage of cell surface pro-N-cadherin-positive tumor cells post-treatment compared to pre-treatment (Table 1), validating our *in vitro* findings. All of these patients received either anthracycline or anthracyline + taxane therapy, and exhibited an incomplete pathologic response. Considering that multiple TN breast cancer subtypes have been identified [27], we hypothesize that cell surface pro-N-cadherin-positive cells may only be enriched in a subset of TN breast cancer subtypes. We further hypothesize that with follow-up, the cases with increased % cell surface pro-N-cadherin-positive cells will exhibit future tumor recurrence. This pilot data underscores the importance of performing a larger, prospective study of pro-N-cadherin expression in TN breast cancer cases pre- and post- neoadjuvant chemotherapy treatment, controlling for TN breast cancer subtype. These follow-up studies have the potential to identify a novel biomarker in a subset of TN breast cancer patients that predicts tumor recurrence. In addition, identifying cell surface pro-N-cadherin as a determinant of chemo-resistance in a subset of TN breast cancers will establish a logical therapeutic strategy for chemo-sensitizing tumors in these patients.

Further studies are needed to determine what drives pro-N-cadherin expression in chemo-resistant TN breast tumor cells. Pro-N-cadherin is cleaved by a specific pro-protein convertase (furin). Reduced furin levels have been reported to promote increased pro-N-cadherin expression in glioma and melanoma cells, resulting in elevated migratory/invasive behavior [12]. Of note, we did not detect reduced furin in chemo-residual TN tumor cells (data not shown), suggesting that alternative signaling drives pro-N-cadherin cell surface expression in chemo-residual TN breast tumor cells. Notably, chemo-residual TN breast tumor cells expressed significantly elevated N-cadherin mRNA levels compared to untreated TN breast tumor cells. Based on this finding, we are currently testing the hypothesis that elevated N-cadherin levels in chemo-resistant cells do not get processed to the mature form because of limiting amounts of the pro-protein convertase, furin.

We are also investigating the mechanisms by which pro-N-cadherin expression increases invasion in TNBC. Possibilities include an ability of pro-N-cadherin to prevent cell-cell adhesion, thus favoring invasion. Alternatively, considering that N-cadherin drives tumor cell invasion/metastasis by associating with FGFR1 and preventing its endocytosis [28, 29], we hypothesize that pro-N-cadherin promotes FGFR1 signaling more efficiently than does N-cadherin, resulting in increased tumor cell invasion.

In summary, our work indicates that TN tumor cells exposed to short-term chemotherapy exhibit increased invasive behavior relative to the untreated tumor cells. We also identify a precursor form of an adhesion protein, the expression of which is upregulated on chemo-resistant TN tumor cells. Considering that the establishment of distant tumor recurrence is highly dependent on chemo-residual tumor cell invasion, we suggest that this precursor adhesion protein may be a central determinant of TN breast cancer recurrence, a topic of current investigation.

## CONCLUSIONS

TN tumor cells surviving short-term chemotherapy treatment exhibit increased invasive behavior compared to untreated tumor cells due to their increased expression of cell surface precursor N-cadherin. Cell surface precursor N-cadherin is expressed on chemo-residual tumor cells from TNBC patients, suggesting the importance of investigating: 1) whether cell surface pro-N-cadherin in TN tumor cells predicts future tumor recurrence, and 2) if this cell surface protein can be targeted to eliminate chemo-residual TNBC disease/prevent recurrence.

## MATERIALS AND METHODS

### Cell culture

SUM159 and BT549 triple-negative breast tumor cells were obtained from Duke Cell Culture Facility in 2010. Both cell lines were authenticated (August 2015) with STR profiling at the Duke DNA facility using GenePrint 10 kit (Promega). SUM159 cells were maintained in Ham's F-12 medium containing 5% heat-inactivated FBS, 5 μg/L insulin, and 1μg/mL hydrocortisone. BT549 cells were maintained in RPMI 1640 containing 10% heat-inactivated FBS, 1 μg/mL insulin, 10 mM HEPES,1 mM pyruvate, and 2.5 g/L glucose.

### Short-term chemotherapy treatment model

SUM159 or BT549 triple-negative tumor cells were cultured for 2 days in Docetaxel (100 nM). After Docetaxel removal, chemo-residual tumor cells were allowed to recover in drug-free complete medium for an additional 16 d. At this time, colonies emanating from chemo-residual tumor cells were harvested with EDTA and expanded as a monolayer for one passage prior to analysis of chemo-residual tumor cell signaling/invasive behavior. To generate chemo-residual tumor cells exposed to two rounds of docetaxel, cells emanating from round 1 of treatment (described above) were subjected to 2 day docetaxel (100 nM) treatment as above. After docetaxel removal, chemo-residual tumor cells were allowed to recover in drug-free complete medium for an additional 16d. Colonies were harvested with EDTA and expanded as a monolayer, as above.

### Thymidine incorporation assay

Cells were seeded into a 96 well tissue culture plate at a seeding density of 5000 cells/well (x 6). After 12 hours incubation at 37°C (5%CO_2_), [Methyl-3H]-Thymidine (Perkin Elmer; 0.5 μCi/well) was added. After incubation at 37°C (5% CO_2_) for 4 h, medium was removed, and cells were harvested onto glass fiber filters (FilterMat, Skatron Instruments). [3H]-Thymidine incorporation was measured as counts per minute (CPM) using a Tricarb 2100TR Liquid Scintillation Analyzer (Packard). Mean thymidine incorporation from 6 wells (+/- SEM) was calculated for each cell population.

### Matrigel transwell invasion assay

Wells (Costar 3422, 24 well, 8um plate) were coated at 4°C overnight with with BME Pathclear Matrigel (Trevigen 3442-005-01; 5 μg/well). Cells were harvested with 5mM EDTA / HBSS (Gibco), and washed 3 x with 10 ml culture medium+ 0.1% BSA. After counting, cells were seeded at 50000 or 25000 cells in 100μl media + pen/strep + 0.1% BSA into the top chamber of Matrigel transwells (triplicate wells for each cell line). For some experiments (Figure 5C), pro-N-cadherin monoclonal antibody [30] (kindly provided by Dr. James Wahl) or isotype control antibody (mouse IgG1, Sigma) were added with cells to top chambers. 600 μl complete media with serum was added to the bottom chamber of each transwell as a source of chemo-attractant. Plates were incubated at 37°C/ 5% CO_2_. After 4 h plates were removed and the tops of the transwell inserts were wiped with a Q-tip to remove cells. The inserts were fixed with cold (-20°C) Methanol for 10 minutes and then stained with 0.2 mg /ml Crystal Violet in 2% Ethanol for 10 min. Inserts were left to air dry overnight and photographed at 100X. The number of invaded cells from 5 fields per insert were counted using cell count in Image J software (NIH). Mean # invasive cells from triplicate wells (+/- SD) was determined for each cell population.

### Tail vein injection model

Immune compromised Nod Scid Gamma (NSG) mice (*n* = 20) were obtained from an in-house breeding colony (Duke Cancer Center) and divided into two groups (*n* = 10 per group). The first group of mice was injected *via* tail vein with 10^6^ parental tumor cells/mouse. The second group was injected with 10^6^ chemo-residual cells/mouse. Subsequent growth of metastatic colonies was monitored over time by *in vivo* bioluminescent imaging. 34 days post-graft, mice were euthanized, and lungs were fixed and removed for quantification of macroscopic metastatic burden. Median number of metastatic nodules was determined for each group.

### Tumor initiating and growth model

NSG mice were divided into eight groups (*n* = 10 mice/group). Cells (pre-chemo or post-chemo) were grafted orthotopically into the inguinal mammary fat pad of NSG mice at dilutions of 10^5^, 10^4^, 10^3^, or 10^2^ cells per mouse. Resulting tumors were tracked by direct caliper measurements.

### Mammosphere culture

Cells were seeded into Mammocult media (Stem Cell Tech., #05620) supplemented with 1% Methylcellulose (Sigma #M0430), pen / strep (Gibco), Heparin (Stem Cell Tech., #07980; 4 μg/mL), and Hydrocortisone (1.0 μg/ml). Sphere assays were setup in Costar 6 Well Ultra Low Attachment (#3471) plates in triplicate. Cells were seeded (20000 cells/well) into each well in complete Mammocult media and incubated at 37°C in 5% CO_2_. Number of spheres (≥ 50 μm) was counted after 7 d using Gel Count. Data were reported as number of spheres from 3 wells (+/- SEM). For secondary spheres, primary spheres were trypsinized, washed with regular media, and seeded at 20000 cells/well as above. Sphere counts were determined on d7, as above.

### Cytosolic and nuclear protein extraction

Cells were harvested using 2mM EDTA / HBSS and washed 2X with HBSS (Gibco). Cytosolic extracts were prepared using lysis buffer [10mM Hepes, pH 7.6, 10mM KCl, 1.5mM MgCl_2_, 0.5% NP40, Halt Phosphatase / Protease Inhibitor (Pierce), PMSF (1mM)]. Cells were lysed on ice for 20 minutes then centrifuged at 3500 rpm for 5 minutes at 4°C. Supernatant containing cytosolic proteins was removed and stored at -80°C. Nuclear proteins were extracted from the pellet on ice for 15 min using nuclear extraction buffer [1% SDS in 50mM Tris pH 7.5, Halt Phosphatase / Protease Inhibitor, 1mM PMSF, 0.5ul Benzonase (Sigma)]. Extracts were centrifuged at 4°C at 14000 rpm for 10 min. Supernatant containing nuclear proteins was removed and stored at -80°C. Protein concentrations were determined using BCA Protein Assay Kit (Pierce).

### Western blotting

1X Gel Loading Buffer with beta mercaptoethanol (5mM) was added to 40 ug of lysate. Samples were boiled for 5 min, and then loaded into a 10% NuPage Bis-Tris Gel (Invitrogen). Proteins were transferred to nitrocellulose in cold 1X Tris/Glycine/10% Methanol with an ice pack at 0.3 amps for 50 min. Membranes were then blocked with Rockland Blocking Buffer (Rockland) for 1 hour. Membranes were probed with primary antibodies overnight at 4c with rocking. Membranes were then washed once with PBS/0.5% Tween 20. Fresh Blocking Buffer was added and IR-dye-conjugated secondary antibodies were added at room temperature with rocking for 40 to 50 minutes. Membranes were washed 3x with PBS/0.5% Tween 20 and 3x with PBS. Proteins were visualized with an Odyssey Infrared Imaging System. Bands were quantified using Image J software (NIH).

### Pro-N-cadherin sorting

SUM159 cells were harvested with 2 mM EDTA in HBSS. Harvested cells were washed in wash buffer (HBSS/0.5% BSA, Pen/Strep), and then incubated with PE-conjugated Pro-N-cadherin (20μl / 2x10^-6^ cells) for 45 minutes at 4°C. Fifteen million cells were typically stained with Pro-N-cadherin-PE antibody. Cells were washed in wash buffer, resuspended in complete media, and placed through a 30 μm cell strainer to obtain single cells. 7-AAD (5 μL per million cells) was added immediately before cell sorting. Live cells (7-AAD negative) were sorted by pro-N-cadherin intensity (top 30% = high and bottom 30% = low/negative).

### Pro-N-cadherin IHC

TN breast cancer patients treated with neoadjuvant chemotherapy that exhibited an incomplete pathologic response were identified from medical records under Duke Institutional Review Board approval (Protocol 47289). Retrospectively collected tumor biopsies (obtained pre- chemotherapy) and biopsies/resections (obtained post-chemotherapy) from these patients were retrieved. Formalin-fixed, paraffin-embedded tissues were subjected to pro-N-cadherin immunohistochemistry. Slides were baked at 60°C for 1 hour, and deparaffinized in xylene followed by 100% ethanol. Antigen retrieval was performed in subboiling Citrate buffer at 100° C for 40 min. Pro-N-cadherin staining was performed in an autostainer according to the following program: Endogenous Peroxidase Quench (R&D-Peroxidase), 5 min; Protein block (R&D Serum block, Avidin block, Biotin block), 15 min each blocking step; Pro-N-cadherin antibody (R&D Systems Sheep anti-pro-N-cadherin (0.4 μg/mL), 2 hour; secondary detection kit (R&D HRP sheep detection kit with DAB), 45 min; DAB, 7 min; hematoxylin, 1 min; Bluing, 1 min. Slides were then placed in water and dehydrated in ethanol to xylene before adding a coverslip. Formalin-fixed, paraffin-embedded slides of SUM159 breast tumor cells [Pro-N-cadherin(+)] and MCF7 breast tumor cells (Pro-N-cadherin(-)] were prepared as positive and negative controls respectively for Pro-N-cadherin reactivity.

### IHC scoring

Two pathologists (EP, GD) (blinded to patient samples) assigned scores for percent tumor cells positive for cell surface (membrane) pro-N-cadherin staining, as well as intensity of staining (+1, +2).

### Clonogenics assay

Pro-N-cadherin-positive and Pro-N-cadherin-negative populations were sorted from untreated SUM159 cells, seeded into 6 well plates (4 wells per condition) at varying cell densities, and incubated at 37°C, 5% CO_2 _overnight. The next day, culture medium was removed and treatments were added [no treatment, Docetaxel (50nM, 75nM, 100nM and 150nM]. After 48 h, treatments were removed, cells were washed with HBSS, and fresh culture medium (lacking drug) was added. Every three days, culture medium was changed and plates were examined for colony formation. To stain colonies, plates were fixed in 10% acetone /10%methanol solution for 10 min. Colonies were stained with 2% crystal violet for 30 minutes. The plates are then washed with water and allowed to air dry. Plates were imaged on GelCount and colonies were counted with Image J.

## SUPPLEMENTARY MATERIALS FIGURES AND TABLES



## List of abbreviations

TN: triple-negative
TNBC: triple-negative breast cancer
Pro-N-cadherin: precursor neural-cadherin
FGFR1: fibroblast growth factor receptor 1
