# Supplementary material for: Chemotherapy enriches for an invasive triple-negative breast tumor cell subpopulation expressing a precursor form of N-cadherin on the cell surface
Source: Oncotarget. 2016 Oct 28;7(51):84030–42. doi: 10.18632/oncotarget.12767 (PMC5356642; doi:10.18632/oncotarget.12767)
Supplement: Supplementary file 1 [file oncotarget-07-84030-s001.pdf]

Chemotherapy enriches for an invasive triple-negative breast tumor cell subpopulation expressing a precursor form of N-cadherin on the cell surface

Supplementary Material

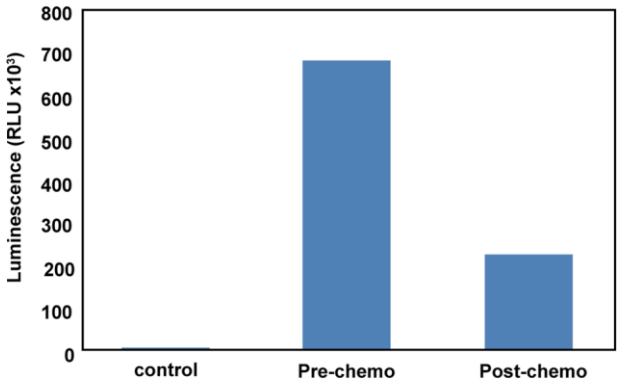

Supplementary Figure 1: *In vitro* luminescence of parental and chemo-residual SUM159 tumor cells. Relative luminescence was determined in equal numbers of parental and chemo-residual tumor cells. Note that chemo-residual cells exhibit reduced luminescence compared to parental cells. Similar results were obtained in 4 independent trials.

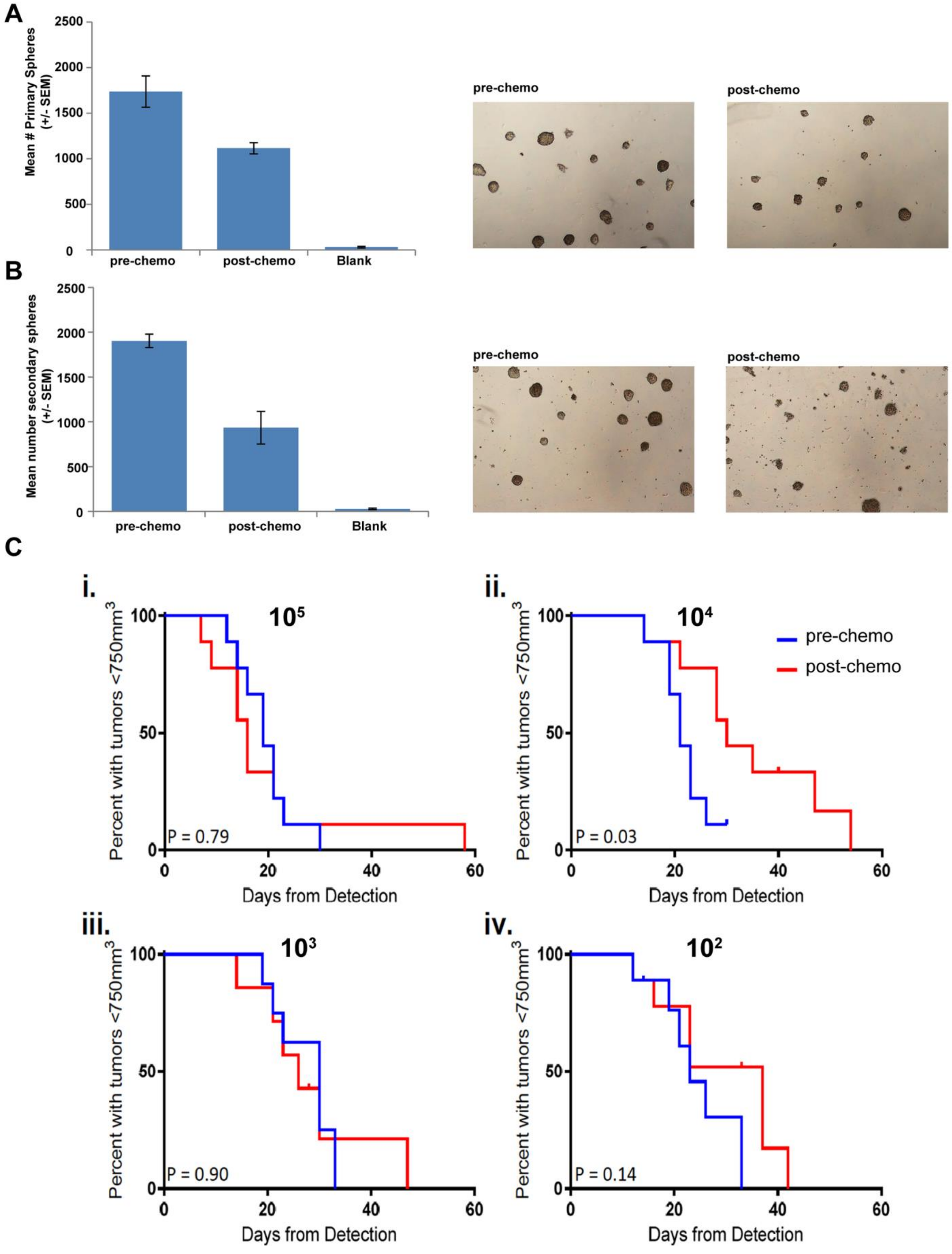

A
Mean # Primary Spheres (+/- SEM)
2500
2000
1500
1000
500
0
pre-chemo
post-chemo
Blank
pre-chemo
post-chemo
B
Mean number secondary spheres (+/- SEM)
2500
2000
1500
1000
500
0
pre-chemo
post-chemo
Blank
pre-chemo
post-chemo
C
i.
10^5
Percent with tumors <750mm^3
P = 0.79
Days from Detection
ii.
10^4
pre-chemo
post-chemo
P = 0.03
Days from Detection
iii.
10^3
P = 0.90
Days from Detection
iv.
10^2
P = 0.14
Days from Detection

Supplementary Figure 2: Chemo-residual TN breast tumor cells derived from a short-term chemotherapy treatment model do not exhibit increased cancer stem-like/tumor initiating activities compared to parental tumor cells. A. Parental and chemo-residual SUM159 cells (harvested on d18 as in Fig. 1) were seeded at equal numbers into a non-adherent mammosphere assay. Number of spheres (≥ 50 µm) was counted after 7 d using Gel Count. Data are reported as number of spheres from 3 wells (+/- SEM) (left panel). Blank well contained no added cells. *, p=0.03, t-test. Representative fields for spheres generated from parental and chemo-residual SUM159 tumor cells are shown in the right panel. Similar results were obtained in 3 independent trials. **B.** Cells from primary spheres generated in A were trypsinized into single cells and seeded at equal numbers into a secondary sphere assay. Spheres were counted as in A. Representative sphere fields are shown in the right panel. **, p= 0.01, t-test. **C.** Parental (blue) and chemo-residual (red) SUM159 cells were injected into the inguinal mammary gland of NSG mice in a dilution series (10^5, 10^4, 10^3, 10^2) (10 mice/group). Mice were monitored for breast tumor growth three times per week. Palpable tumors were measured with calipers. Data is plotted as percent of tumors smaller than 750mm^3 over time. P values, as determined by the log-rank Mantel-Cox test, are indicated. Note that no difference in tumor take was observed between parental and chemo-residual TN tumor cells for any cell injection number.

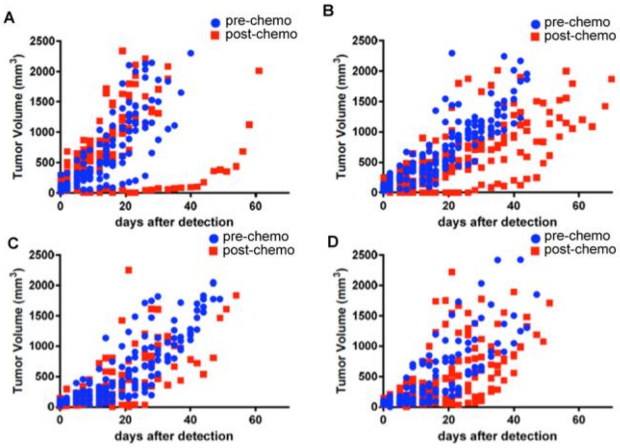

Supplementary Figure 3: Tumor growth rate of parental and chemo-residual SUM159 tumor cells. Tumor growth rate in mice receiving a graft of : A. 10^5, B 10^4, C. 10^3, or D. 10^2 SUM159 parental (blue) or SUM159 chemo-residual (red) tumor cells/mouse.

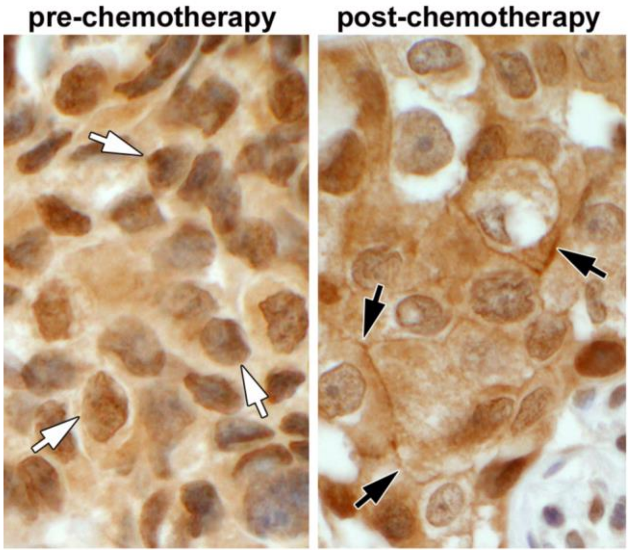

Supplementary Figure 4: Pro-N-cadherin immunohistochemistry on pre- and post-neoadjuvant chemotherapy-treated TN breast cancer cases. Matched cases were obtained from six TNBC patients pre- and post- neoadjuvant chemotherapy treatment. A representative matched case is shown. Note that nuclear/peri-nuclear staining (white arrows) is observed both pre- and post- chemotherapy. However, cell surface pro-N-cadherin staining (black arrows) is only observed post-chemotherapy treatment.
